# Ethnobotanical investigation of 'wild' food plants used by rice farmers in Kalasin, Northeast Thailand

**DOI:** 10.1186/1746-4269-7-33

**Published:** 2011-11-08

**Authors:** Gisella S Cruz-Garcia, Lisa L Price

**Affiliations:** 1 Centre for Crop Systems Analysis, Department of Plant Sciences, Wageningen University, Wageningen, The Netherlands; 2 Department of Social Sciences, Wageningen University, Wageningen, The Netherlands; 3 Department of Anthropology, Oregon State University, Corvallis Oregon, USA

**Keywords:** Wild food plant, ethnobotany, rice ecosystem, edible part, use, growth location, growth form, gathering, Thailand, Southeast Asia

## Abstract

**Background:**

Wild food plants are a critical component in the subsistence system of rice farmers in Northeast Thailand. One of the important characteristics of wild plant foods among farming households is that the main collection locations are increasingly from anthropogenic ecosystems such as agricultural areas rather than pristine ecosystems. This paper provides selected results from a study of wild food conducted in several villages in Northeast Thailand. A complete botanical inventory of wild food plants from these communities and surrounding areas is provided including their diversity of growth forms, the different anthropogenic locations were these species grow and the multiplicity of uses they have.

**Methods:**

Data was collected using focus groups and key informant interviews with women locally recognized as knowledgeable about contemporarily gathered plants. Plant species were identified by local taxonomists.

**Results:**

A total of 87 wild food plants, belonging to 47 families were reported, mainly trees, herbs (terrestrial and aquatic) and climbers. Rice fields constitute the most important growth location where 70% of the plants are found, followed by secondary woody areas and home gardens. The majority of species (80%) can be found in multiple growth locations, which is partly explained by villagers moving selected species from one place to another and engaging in different degrees of management. Wild food plants have multiple edible parts varying from reproductive structures to vegetative organs. More than two thirds of species are reported as having diverse additional uses and more than half of them are also regarded as medicine.

**Conclusions:**

This study shows the remarkable importance of anthropogenic areas in providing wild food plants. This is reflected in the great diversity of species found, contributing to the food and nutritional security of rice farmers in Northeast Thailand.

## Background

The collection and consumption of 'wild' plant foods from agricultural and non-agricultural ecosystems has been documented in multiple cultural contexts, illustrating their use and importance among farming households throughout the world [1-3]. The evidence to date suggests that gathering by farmers occurs in various environments, ranging from intensively farmed areas, to more subsistence oriented horticultural systems, and finally in more pristine areas such as forests. This is certainly the case of rice farmers in Asia [4]. For example, Ogle et al. [5,6] found that in the Mekong Delta of Vietnam 90% of women eat wild vegetables, uncovering a total of 94 species. Kosaka [7], in his research on flora from the paddy rice fields in Savannakhet, Laos, recorded 11 edible species from a total of 19 herbaceous useful plants, and 25 food trees out of 86 useful species. The documentation of 'wild' food plant gathering and consumption in mainland Southeast Asia is still growing, however the literature is scattered across numerous disciplines [8].

The research on which this paper is based was conducted in Kalasin Province, Northeast Thailand. Studies conducted in this region provide documentation that 'wild' food plants are a critical component in the subsistence system of farmers [9-14]. This food resource is extremely important to the rural population comprised of rice farmers, given that the Northeast region is regarded as both Thailand's largest and poorest part of the country. This paper adds to this literature by providing the most comprehensive botanical inventory of these foods to date. Two botanical characteristics are described in this article: growth form and life cycle. Moreover, we present the growth location of the plants. Regarding cultural characteristics, this paper also identifies multiple uses of wild food plants.

Wild food plants in this article refer to non-domesticated plants. These plants exist on a continuum of people and plants interactions in regard to their degree of management. In this way, wild food plants include those from 'truly' wild to wild protected, cultivated and semi-domesticated plants that may be promoted, protected or tolerated in some way locally. Wild food plants can be cultivated, but not all cultivated plants are domesticated. For most species the transition from cultivation to domestication never happens. Human plant management does not necessarily move toward greater intensity and ultimately plant domestication. While some plants are moving towards domestication, other plants that used to be highly managed in the past could be only slightly tolerated and protected under contemporary circumstances [1]. While we include in our definition 'introduced' and 'naturalized' plants, locally domesticated plants are excluded. We use the term 'local' because, since the nature of this study is ethnobotanical, we based our research on these plants that are classified as 'wild' by local people. This is why some food plants that are regarded as 'wild' in Kalasin, might be treated as domesticates in other areas.

### The research site

The research for this paper was conducted in four villages in Kalasin Province, Northeast Thailand. The villages are fairly typical for the region. Kalasin is located at 152 m above sea level (asl) in the Korat Plateau, which geographically defines the Northeast region of the country. This Plateau, forming a shallow depression between 100 m and 200 m asl, is generally quite flat with scattered swamps and ponds (some seasonal) and low hills that rise to around 300 m asl [15].

Soils in this region are mostly heavily leached fine sandy loams, with poor drainage and high salinity. Furthermore they are usually low in phosphate, nitrogen and organic matter [15]. Declining soil fertility is prevalent in the region [16]. Nevertheless, the soils in lowland paddy fields are better than in the uplands because they receive nutrient in-flows eroded from the higher areas [17]. The natural vegetation of this region is dry monsoon forest, primarily composed by dry dipterocarp forest [15,18], with *Dipterocarpus tuberculatus *Roxb., *D. obtusifolius *Teijsm. ex Miq., *Shorea obtusa *Wall., *S. siamensis *Miq., *Xylia xylocarpa *(Roxb.) Taub., *Irvingia malayana *Oliv. ex A.M. Bennett, *Cratoxylon formosum *(Jack) Dyer. and *Careya arborea *Roxb. as dominant species [19].

Deforestation has been occurring at a high rate since the early 1950s with the extension of agricultural land due to commercialization of agriculture, as well as population growth. In this way, the forest and wooded areas have decreased from 90% in the 1930s to less than 14% in 2004. The rate of deforestation was likely augmented significantly during the economic crisis at the end of the 1990s [16,20]. At the same time, soil degradation in the agricultural areas has been increasing and consequently yields have declined [17].

The Northeast covers 170, 000 km^2 ^[17] and has more land dedicated to agriculture than the rest of the country (9.25 million hectares). Around 94% of the region's population live in rural areas [21], with the region possessing the highest number of farms in the Nation (2, 273, 000) [22]. Indeed, in Kalasin province 85.1% of the population depend on agriculture [23]. The main crop is glutinous rice (also called sticky rice), which is important as the dietary staple and for income generation. Rice production corresponds to 70% of the arable land of the Northeast, but average rice yields are the lowest in the country (1.8 Mg ha^-1^) [16]. Within the traditional rain-fed paddy agricultural system, which is primarily transplanted rice, crops can be damaged by delayed rains when transplanting seedlings, or by droughts and floods [15,16]. The annual monsoon provides 90% of the annual rainfall of the Northeast, averaging over 200 mm from May through October, which is essential for the cultivation of glutinous rice. From November to April, rainfall averages only about 20 mm per month in Kalasin [24].

### The research population

The Northeast is referred to as *Isaan *and is also known for its distinct cultural characteristics. The people who inhabit the region, commonly referred to as *Isaan *people, are ethnically of Lao origin, constituting one of the largest minority populations in the country. Most Northeasterners speak a dialect of Lao mixed with some influences from Thai also known as *Isaan*. *Isaan *is written using the Thai script. Thai is learned formally in school and villagers are literate in Thai, except for the very elderly.

Kalasin Province has a population of about one million inhabitants and a density of 132.3 inhabitants/km^2^. Households on average have four family members in the rural areas, and 23.6% of them are female headed. Theravada Buddhism is the main religion in this province (99.5% of the population), as in the rest of the country. The population has attained on average 6.5 years of education. Regarding their work status, 51.7% are unpaid family workers and 35.8% are engaged in self-employment, usually in agriculture [23]. There is a high rate of seasonal or full-time migration to major cities mainly as wage labourers who aim to send remittances to their families that stay in the rural areas [19]. Off-farm employment accounts for two thirds of the total income of families in Northeast Thailand [21].

There is customary inheritance of land through women and a pattern of matrilocal residence. This system facilitates women having a thorough knowledge of their social and physical environment [8].

### General overview of wild food plants in Northeast Thailand

An important yet not widely available study at the national level established that wild food plants play an essential role in the diet in all the rural areas of Thailand [25]. This is clearly reflected in the fact that more than 500 different edible natural products have been documented as being sold in the markets around the country [26].

Gathering mainly occurs in anthropogenic ecosystems, such as agricultural lands (including paddy fields), woody areas, (home) gardens, house areas and swamps [12,14,27]. Agricultural lands and home gardens are traditionally owned by women [28-30]. In Northeast Thailand, women are the main gatherers, selectors, transplanters and propagators of wild food plants [27-33].

In this region farmers have as their staple glutinous rice accompanied by a variety of wild foods derived from wild, semi-domesticated and domesticated plants, as well as frogs, paddy crabs, insects and fish. During the rainy season wild food can constitute as much as half of the total food consumed in the villages. Wild food plants are mainly consumed as fresh fruits or vegetables eaten raw or steamed, and in local "curries" or soups [34,35].

In fieldwork conducted in Northeast Thailand in 1990, Price documented 77 species gathered by farmers in a village in Kalasin Province [14,36]. Somnasang, Rathakette and Rathanapanya [34] listed 42 wild vegetables and 7 wild fruits in a paper published in the 1980s. Ten years later, Somnasang, Moreno-Black and Chusil [33] recorded 66 wild food plants consumed in Northeast Thailand. Furthermore, Sapjareun, Kumkrang and Deewised [37] published a book, in Thai, entitled "Local vegetables in *Isaan*" presenting a general description of a number of plants by species, their propagation, ecological importance and uses, as well as the local recipes.

### The botanical-dietary paradox

One of the important characteristics of wild plant foods among farming households is that the main collection locations are increasingly from the anthropogenic ecosystems such as agricultural areas rather than pristine ecosystems [14]. Ogle and Grivetti [38] in their study in Swaziland found that the most intensively cultivated area among their research sites exhibited the highest level of loss of edible species, but, at the same time, the most consumption of wild food plants. They termed this phenomenon the "botanical-dietary paradox" and proposed that this occurs when people start to rely on eating the weeds of agriculture once a decline in forests occurs. Ultimately, the species that are considered local vegetables change. Price and Ogle [8] further explain that time constraints are a major factor in the commencement of the botanical-dietary paradox in that as forests decrease and become more remote from the village, gathering from the forests becomes increasingly too time consuming, so farmers shift to gathering in areas closer to home and shift to eating many of the weeds of agriculture and other food plants in the agricultural system. This shift in food resources is evident on Mainland Southeast Asia.

Saowakontha et al. [39] conducted a study on edible forest products in two villages, Ban Moh and Ban Nong Khong, Phu Wiang district, in Northeast Thailand, presenting a list of 34 wild food plants. They found that the degree of dependency on this resource was related to the distance from the village to the forest, thus, the longer the distance to the forest, the higher the dependency on other areas for food gathering. Likewise, Kosaka et al. [40,41] compared two rice farming villages from Savannakhet Province, Laos, obtaining the same results. Whereas Bak village, located in the uplands with an extensive forest area, showed to be more dependent on forest diversity, farmers from Nakou village, situated in the lowlands with a small area of remnant forest, identified more useful plants from the rice fields than the forest, compensating for the lack of resources by maintaining the tree diversity within the paddy rice fields. Studies conducted specifically on non-timber forest products provide surprising results. For example, in a study conducted in the Lao P.D.R., the researchers discovered that farmers used multiple land types and that 60% of the *non-timber forest products *were not from the forest at all but were collected from fields (paddy, dry grass areas, and fallow), streams and ponds [42]. The same happened when Shibahara conducted research on hunting and gathering in public forests of Roi Et, Northeast Thailand. Although research was focused on forest areas, a major finding was that farmers relied mainly on wild foods from rice fields rather than forests. Shibahara also emphasized that most gathering activities occurred on private land instead of public land [43]. The role of private land in food gathering entitlements among Northeast Thai villagers has been documented by Price [14].

Given the alarming rate of decrease in forest and wooded areas in Thailand [44] it is becoming increasingly important to also study the wild food plants from anthropogenic areas, as several studies have shown that farmers are becoming more dependent on these places for ensuring their household dietary diversity and food security [8,12,14,45,46].

Somnasang, Rathakette and Rathanapanya [34] found that paddies are a principal place for gathering wild vegetables and fruits in Northeast Thailand. Likewise, Price [14] estimated that farmers gather more from the fields than from any other place. Indeed, rice fields are not only important in terms of rice production but are biologically diverse [47] and multi-resource agro-ecosystems [9]. According to the International Rice Research Institute [4], paddies possess over 100 useful associated plant species being sources of food, medicine, fibre, construction material, fuel and animal feed.

### Anthropogenic ecosystems

Rice fields on the plains of Northeast Thailand and Laos are characterized by having trees in the paddy fields, given their importance for local culture [40] and their socio-economic and ecological functions [48]. Trees are either planted or remnants from a previous forest, which went through different stages of transformation until becoming a rice field during the historical and on-going process of agricultural expansion [9,17,18]. The transition point was named "rice production forest" by Takaya and Tomosugi [49]. Vityakon et al. [17] recognize different transitional historical stages of land use change, which they describe at the regional, community, landscape and field level in their article "From forests to farm fields: changes in land use in undulating terrain of Northeast Thailand at different scales during the past century". Prachaiyo [18] also explains this process in his publication entitled "Farmers and forests: a changing phase in Northeast Thailand".

There are a number of studies on the diversity of trees in paddy fields in Northeast Thailand. Grandstaff, Rathakette, and Thomas [9] recorded 54 species of trees and shrubs, 32 of them used as food and/or medicine, growing in the rice fields. Watanabe et al. [50] recorded 16 useful tree species growing in paddy fields in the region. Additionally, Vityakon [48,51-53] conducted research on the importance of trees for soil fertility in rice fields. She identified 25 species (14 of them used as food and/or medicine) surviving from previous forests, indicating, if applicable, their uses as food and/or medicine [51]. Later on, Prachaiyo [18] described 28 useful tree species growing in the paddies mainly for timber, latex, food, medicine, oil or fodder. Subsequently, Tipraqsa [19] emphasized the importance of trees in rice fields in Northeast Thailand, documenting 52 trees found in the diverse farming systems in the rice landscape. Finally, trees in rice fields have also been systematically documented in Laos by Kosaka et al. [7], and also discussed in the symposium "Tree-Rice Ecosystem in the Paddy Fields of Laos" organized by a Japanese-Thai project on the same topic, where the utilization of some tree species as food was noted [54].

Plant diversity in rice fields not only consists of trees, but also aquatic and terrestrial herbs, climbers and shrubs. However, several herbs, climbers and shrubs are classified as weeds or invasive species by agronomists. Yet, a number of weeds are used as vegetables or medicines in Thailand. Maneechote [55] documents 59 edible weeds indicating their parts eaten and the habitat where they grow, which corresponds to about 30% of the 150 plant species classified as weeds in the country. Vongsaroj and Nuntasomsaran [56] conducted a literature review on weed utilization in Thailand reporting 33 weeds used as food, 16 as medicine and 12 as animal feed; some of them were also listed later on in Vongsaroj's [57]. Kosaka et al. [41] identified 11 edible species, 5 medicinal species and 2 plants used as animal feed, mostly weeds from the paddy fields in Savannakhet, Laos.

Prachaiyo also listed some herbs used as vegetable or medicinal plants growing in the rice fields of Northeast Thailand [18]. Although weeds have been shown to have diverse uses around the world [58], they are continuously overlooked in their role as sources of food and medicine [55]. Minor attention is paid to weed utilization in Thailand given that most agricultural research is focused on minimizing their population [56].

### This study

Despite the recognition of the important role that wild food plants play for farmers' livelihoods in Northeast Thailand, information is rather scattered throughout different publications, which are mainly in the Thai language. There is no single article presenting not only an exhaustive list of species but also their local name and, botanical and cultural characteristics. This is certainly necessary as a baseline for future research in this area.

The objectives of this paper are to provide selected results from an ethnobotanical study of wild food plants conducted in Northeast Thailand. A complete botanical inventory of wild food plants used by the study villages and their surrounding areas is provided including their diversity of growth forms, the different anthropogenic locations were these species grow and the multiplicity of uses they have. The research presented in this paper contributes to understanding the importance of different anthropogenic ecosystems where wild food plants grow and provides insights on the multiplicity of uses of these plants.

## Methods

### Taxonomic identification and plant naming

Fieldwork was conducted from 2006 to 2010, taking as a baseline the results obtained in research carried out by one of the authors in two adjacent villages located in Kalasin Province, where she identified 77 species classified as 'wild' food plants during focus group elicitations conducted with local farmers [14]. This list was built upon and increased using focus groups and key informant interviews as complementary methods in the same villages. A final list of 87 species of locally classified 'wild' food plants was constructed and local names of plants in the local Thai-Lao vernacular were recorded in the Thai script. Species were botanically identified by taxonomists from the Department of Biology of Chang Mai University and Walai Rukhavej Botanical Research Institute of Mahasarakham University. Herbarium specimens of most of the identified species are on repository in one or more locations in Thailand, including the Bangkok Herbarium of the Department of Agriculture (BK) in Bangkok, Herbarium of Walai Rukhavej Botanical Research Institute (WRBG) in Mahasarakham, and the Herbarium of Khon Kaen University (KKU) in Khon Kaen. Botanical naming of family, genus and species follows "Flora of Thailand" [59].

The villagers use the term *geht eng*, which means "birth itself" for wild food plants. However they do distinguish between "birth itself" as a type of plant versus just the verb "to birth by itself" (without human intervention, such as sowing or transplanting). Some "birth itself" species can also be transplanted or propagated, as some domesticates such as tomatoes can "birth themselves" (growing from consumption debris). Domesticates that "birth themselves" are not considered wild food plants ("birth itself" *type *of plant). Plant types are further identified by prefixes. The most common prefixes used for naming food plants refer to their edible part, such as *bak *and *maak *that mean fruit *yod *meaning shoots *bai *(which is a more unusual prefix) referring to leaf  and *dok *that means flower  A very common prefix for naming wild food plants is *phak *which means vegetable [14]. *Phak *includes shoots, leaves, stems and sometimes whole aerial parts eaten as vegetable. In this way, if a plant has more than one edible part, it will likely have more than one name differing in the prefix used. For example, *Garcinia cowa *has two local names Phak moong  and Bak moong  given that it is eaten as both vegetable and fruit. A total of 131 plant names were documented for the 87 plants, giving an average of 1.5 names per plant. Plant names were carefully recorded in the local *Isaan *dialect (capturing both pronunciation and local tone) using the Thai script. Plant names were also transliterated into English. Finally, English names were obtained from Germplasm Resources Information Network (GRIN) [60], Multilingual Multiscript Plant Name Database (MMPND) [61] and Plant Resources of Southeast Asia (PROSEA) [62].

### Ethnobotanical data collection

Growth form and life cycle were determined for each species through field observation and complemented with literature [63]. Growth location and cultural characteristics of the plants, such as edible parts and multiple uses, were assessed through focus groups and supplemented with key informant interviews conducted not only in the research villages but also in two additional nearby villages. The use of different methods permitted, to a certain degree, triangulation and greater depth. These activities were carried out with the aid of local translators who speak the Thai-Lao vernacular of the Lao language (*Isaan*) as it is spoken in the research location and are knowledgeable about the research topic. Finally, a relational data base of wild food plants was built using Microsoft^® ^Access.

Focus groups are particularly useful when the everyday use of language and culture of particular groups is of interest, and when one wants to explore the degree of consensus on a given topic [64]. The focus group method has previously been successfully applied to the collection of plant species level information with farmers in Northeast Thailand [14]. Each focus group consisted of six to nine members, following Bernard's recommendations on the number of participants [65]. Focus group participants were generally middle-age women or slightly older (34 to 66 years old), named by the villagers themselves as knowledgeable about contemporarily gathered plants [65,66]. A total of 12 sessions were carried out sometimes with different participants, each session lasted two to three hours and was tape recorded. All of who participated in the study did so freely and with consent.

## Results and discussion

### Botanical characteristics of wild food plants

A total of 87 wild food plants, belonging to 47 families, were mentioned by farmers through key informant interviews and focus group discussions in 2006, building up on a previous list of plants documented by Price in 1990. Out of this total, 76 plants were botanically identified to the species level recognizing a total of 75 different species (two plants correspond to different sub-species of the same species), 9 were identified to genus level and for two botanical identification was not possible (Table 1). About 13% of the plants were from the Leguminoseae family (6 species belonged to Mimosoideae and 5 to Caesalpinioideae). Other important families were Annonaceae, Myrtaceae, Poaceae, Pontederiaceae, Sapindaceae, Zingiberaceae, with 3 species each.

**Table 1:** List of wild food plants

Scientific name	English transliteration of local (Isaan) name	Local (Isaan) name	English name
**Aizoaceae**			

*Glinus oppositifolius *(L.) Aug.DC.	Phak kaen khom		

**Amaranthaceae**			

*Amaranthus viridis *L.	Phak hom		green amaranth, pigweed, slender amaranth

**Anacardiaceae**			

*Mangifera caloneura *Kurz	Bak muang paa		

*Spondias pinnata *Kurz	Bak kawek		common hog plum, Indian mombin, Andaman mombin

**Annonaceae**			

*Polyalthia debilis *Finet & Gagnep.	Bak lok kok		

*Polyalthia evecta *Finet & Gagnep.	Bak tong leeng		

*Uvaria pierrei *Finet & Gagnep.	Bak pii puwen		

**Araceae**			

*Amorphophallus *sp.	Phak e-loke		

**Araliaceae**			

*Irvingia malayana *Oliver	Bak bok		barking deer's mango
	Maak bok		

**Arecaceae**			

*Borassus flabellifer *L.	Bak taan		palmyra palm, tala palm, wine palm
	Yod taan		

*Calamus *sp.	Bak waai		
	Waai		

**Asclepiadaceae**			

*Telosma minor *Craib	Phak kik		
	Dok kik		
	Bak kik		

**Basellaceae**			

*Basella rubra *L.	Phak pang		Ceylon-spinach, Malabar-nightshade, vine-spinach

**Bignoniaceae**			

*Dolichandrone serrulata *Seem.	Kee paa		

*Oroxylum indicum *Vent.	Phak lin faa		midnight horror, oroxylum
	Bak lin faa		
	Yod lin faa		
	Bai lin faa		

**Burseraceae**			

*Canarium subulatum *Guillaumin	Bak luwam		

**Campanulaceae**			

*Lobelia begonifolia *Wall.	Phak luem phua		

*Lobelia *sp.	Phak som		

**Clusiaceae**			

*Cratoxylum formosum *(Jack) Benth. & Hook.f. ex Dyer	Phak tew		

*Garcinia cowa *Roxb.	Phak moong		cowa
	Bak moong		

**Compositae**			

*Blumea balsamifera *DC.	Phak naad		

*Emilia sonchifolia *(L.) DC.	Phak lin pii		emilia, sow thistle

**Convolvulaceae**			

*Cuscuta chinensis *Lam.	Phak mai tong		Chinese dodder

*Ipomoea aquatica *Forssk.	Phak bung		Chinese water-spinach, swamp morning-glory

**Cucurbitaceae**			

*Coccinia grandis *(L.) Voigt	Phak tam nin		ivy gourd, little gourd
	Bak tam nin		
	Tam nin		

*Momordica charantia *L.	Phak sai		balsam-apple, bitter gourd, bitter melon
	Bak phak sai		

**Ebenaceae**			

*Diospyros rhodocalyx *Kurz	Maak koo		

**Euphorbiaceae**			

*Phyllanthus acidus *(L.) Skeels	Bak yom		gooseberry-tree, Indian-gooseberry, star-gooseberry
	Yod bak yom		

**Fagaceae**			

*Castanopsis *sp.	Bak kaaw		

**Gnetaceae**			

*Gnetum *sp.	Bak muway		

**Hydrocharitaceae**			

*Ottelia alismoides *(L.) Pers.	Phak hob hep		duck-lettuce, water-plantain ottelia

**Hydrophyllaceae**			

*Hydrolea zeylanica *(L.) J.Vahl	Phak ka-liang		Ceylon hydrolea

**Lauraceae**			

*Cassytha filiformis *L.	Phak mai		dodder-laurel

**Lecythidaceae**			

*Barringtonia acutangula *(L.) Gaertn.	Phak kadon naam		Indian-oak
	Kadon naam		

*Careya arborea *Roxb.	Phak kadon kok		
	Kadon kok		

**Leguminosae**			

*Adenanthera pavonina *L.	Phak lam		coralwood, red sandalwood-tree

*Cajanus cajan *(L.) Millsp.	Bak tua heea		pigeon-pea, red gram
	Tua heea		

*Cassia siamea *Lam.	Phak khee lek		kassodtree, Thai cassia, Siamese senna
	Khee lek		

*Dialium cochinchinense *Pierre	Bak keng		velvet-tamarind

*Leucaena leucocephala *(Lam.) de Wit	Phak kased		leadtree, white popinac, leucaena
	Bak kased		
	Yod phak kased		
	Kased		

*Neptunia javanica *Miq.	Phak kased kok		

*Neptunia oleracea *Lour.	Phak kased naam		water-mimosa

*Pithecellobium dulce *(Roxb.) Benth.	Bak kaam lian		blackbead, Manila tamarind, sweet-inga
	Kaam lian		

*Senna sophera *(L.) Roxb.	Phak let ket		Kasondi senna

*Sindora siamensis *Teijsm. ex Miq.	Bak tee		

*Tamarindus indica *L.	Bak kaam		tamarind
	Bak kaam som		
	Maak kaam		
	Yod kaam		

*Xylia xylocarpa *Taub. var *kerrii *(Craib & Hutch) I.C. Nielsen	Bak deeng		
	Maak deeng		

**Liliaceae**			

*Asparagus racemosus *Willd.	Phak shi shang		Indian asparagus
	Shi shang		

**Limnocharitaceae**			

*Limnocharis flava *Buchenau	Phak kanjong		sawah-flower rush, sawah-lettuce, velvetleaf
	Bak kanjong		
	Phak pai		

**Marsileaceae**			

*Marsilea crenata *C.Presl	Phak waen		pepperwort, water clover

**Meliaceae**			

*Azadirachta indica *A.Juss. var. *indica*	Phak ki nin		sadao India

*Azadirachta indica *A.Juss. var. *siamensis *Valeton	Phak kadaw		sweet neem, Thai neem
	Yod kadaw		
	Yod phak kadaw		

**Menispermaceae**			

*Cissampelos pareira *L.	Bai maa noi		velvetleaf
	Maa noi		

*Tiliacora triandra *Diels	Yaa nang		
	Bai yaa nang		

**Menyanthaceae**			

*Nymphoides indica *(L.) Kuntze	Phak kanong ma		banana-plant, water-snowflake

**Moraceae**			

*Artocarpus lacucha *Roxb.	Bak haad		monkey-jack, monkeyfruit
	Maak haad		

**Myrtaceae**			

*Psidium guajava *L.	Bak sidaa noi		guava

*Syzygium cumini *(L.) Skeels	Bak waa		jambolan, Java-plum, Malabar-plum

*Syzygium gratum *(Wight) S.N.Mitra	Phak mek		
	Maak mek		

**Nymphaeaceae**			

*Nymphaea pubescens *Willd.	Phak sai bua		red water-lily
	Sai bua		

**Onagraceae**			

*Ludwigia adscendens *(L.) H.Hara	Phak phee phui		water-primrose

**Opiliaceae**			

*Melientha suavis *Pierre	Phak waan paa		melientha

**Passifloraceae**			

*Adenia viridiflora *Craib	Bak saap		
	Phak saap		

*Passiflora foetida *L.	Tam nin farang		running pop, stinking passionflower, wild water-lemon

**Poaceae**			

*Bambusa *sp.	Naw mai phai huwak		

*Bambusa bambos *(L.) Voss	Naw mai phai paa		giant thorny bamboo, spiny bamboo

*Vietnamosasa ciliata *(A.Camus) T.Q.Nguyen	Naw jood		

**Pontederiaceae**			

*Eichhornia crassipes *(Mart.) Solms	Phak katok		water-hyacinth
	Phak paud		

*Monochoria hastata *(L.) Solms	Phak top		arrow-leaf monochoria, hastate-leaf-pondweed
	Phak top thai		

*Monochoria vaginalis *C.Presl	Phak e-hin		oval-leaf monochoria, pickerel-weed

**Rhamnaceae**			

*Ziziphus mauritiana *Lam.	Bak tan noi		Indian jujube, Indian plum, Sour jujube

*Ziziphus oenoplia *(L.) Mill.	Bak lep meuw		jackal jujube, small-fruited jujube, wild jujube
	Maak lep meuw		

**Rubiaceae**			

*Oxyceros horridus *Lour.	Bai kat kaaw		

*Rothmannia wittii *(Craib) Bremek.	Bak maaw		

**Rutaceae**			

*Aegle marmelos *Corrêa	Bak tuum		bael, belfruit-tree, golden-apple
	Maak tuum		
	Yod maak tuum		

**Sapindaceae**			

*Lepisanthes rubiginosa *(Roxb.) Leenh.	Bak huat kaa		rusty sapindus

*Nephelium hypoleucum *Kurz	Bak ngeuw		
	Maak ngeuw		

*Schleichera oleosa *(Lour.) Oken	Bak kawe		Ceylon-oak, lactree
	Luk kawe		
	Maak kawe		

**Scrophulariaceae**			

*Limnophila aromatica *Merr.	Phak kayang		swampleaf

**Umbelliferae**			

*Centella asiatica *(L.) Urb.	Phak nok		Asiatic pennywort, pennyweed, sheep-rot

*Oenanthe javanica *DC.	Phak shi naam		Chinese-celery, Indian pennywort, water-celery

**Zingiberaceae**			

*Alpinia malaccensis *C.Presl	Kaa paa		

*Curcuma singularis *Gagnep.	Dok ka-jeeuw		

*Curcuma *sp.	Dok waun		

**Zygnemataceae**			

*Spirogyra *sp.	Taw		

**Unidentified**			

sp. 1	Phak muad		

sp. 2	Phak pe		

Botanical family, scientific name, local *Isaan *name(s), English transliteration of local *Isaan *name(s) and English name(s).

**Figure 1:**
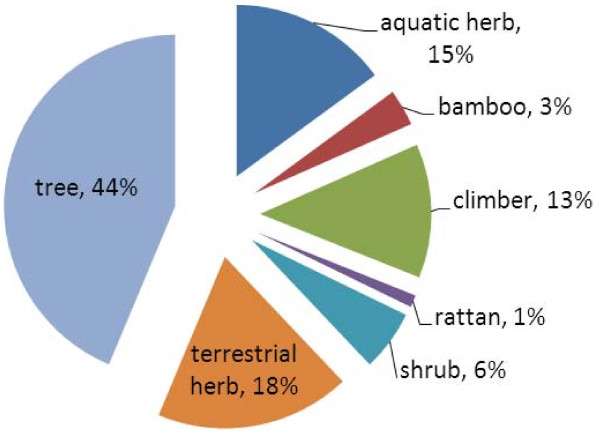
**Growth forms of wild food plants**.

Two categories of life cycles were considered: annual and perennial. Some 79% of the wild food plants were perennial and 21% annual. For analysing growth form, seven categories were considered: aquatic herb, terrestrial herb, climber, shrub, tree, bamboo and rattan. Figure 1 shows that almost half of the wild food plants were trees (44%). Other important growth forms were terrestrial herb (18%), aquatic herb (15%) and climber (13%). Shrubs only presented five plants, followed by bamboo with three plants and rattan with only one plant. Climber and terrestrial herbs were both annual and perennial, while aquatic herbs were only annual plants. Trees, shrubs, bamboos and rattans were all perennial plants.

### Growth location of wild food plants

From an ecological perspective, local farmers provided two major kinds of answers when they were asked where a plant grows. Firstly, (a) they gave general names of what ecologists regard as anthropogenic ecosystems, such as rice field or home garden; and secondly (b) they provided names of specific sub-systems of an anthropogenic ecosystem, such as field margin, tree row or water pond, which all are part of the rice ecosystem. In order to facilitate the analysis, the answers were grouped into six major growth locations: rice field, secondary woody area, home garden, upland field, swamp and roadside, including plants that grow in any of the sub-systems. The analysis of the ethnoecological classification of growth locations (local *emic *categorization) was not an objective of this paper. The six major growth locations of wild food plants are the following:

1. Rice field, containing a diverse range of aquatic, semi-terrestrial and terrestrial niches, is where most wild food plants, roughly 70%, can be found. Only six plants out of 61 are exclusively found in the rice fields (mainly terrestrial herbs regarded as weeds), whereas the rest can also be found in other places, mostly home gardens (64%), secondary woody areas (45%), upland fields (40%) and swamps (20%). In rice fields it is possible to find aquatic herbs such as *Nymphaea pubescens *and *Neptunia oleracea*; terrestrial herbs such as *Limnophila aromatica *and *Amaranthus viridis*; trees as *Borassus flabellifer *and *Leucaena leucocephala*; and climbers like *Coccinia grandis*.

2. Fifty-five percent of the plants occur in secondary woody areas, which are mainly public areas located outside the farms, near upland fields. Only eight out of 48 plants were noted as growing exclusively in woody areas, whereas the rest grow also in other locations, mainly rice fields (68%) and/or home gardens (65%), some of which having been transplanted by the villagers. Most of the wild food plants growing in the woody areas are trees (65%), such as *Azadirachta indica *(also growing in home gardens and rice fields) and *Canarium subulatum *(found only in woody areas). A culturally important terrestrial herb only gathered in woody areas is *Curcuma singularis*, which is gathered in the rainy season.

3. Fifty-two percent of the plants occur in home gardens. There were no plants exclusive to home gardens, all plants could be found in other locations, mainly rice fields (78%), woody areas (58%) and upland fields (49%). Many species growing in home gardens are transplanted from other areas and subject to different degrees of management, such as *Tamarindus indica*. Species in home gardens are mostly trees (e.g. *Phyllanthus acidus*) and climbers (e.g. *Tiliacora triandra *and *Momordica charantia*), followed by a few terrestrial herbs (e.g. *Centella asiatica*).

4. Upland fields, mainly consisting of fields with cash crops of cassava and sugar cane, contain 37% of the wild food plant species. No plants were exclusive to the upland fields. Wild food plant species that occur in upland fields also grow in other locations, mainly woody areas (84%), rice fields (69%) and home gardens (69%). Most species are trees such as *Syzygium gratum *and *Careya arborea*.

5. Swamps contained 17% of the plants. Three out of 15 plants were exclusive to swamps, but these are rarely found. The rest of the plant species also occur in rice fields, with the exception of *Neptunia javanica *which is a terrestrial herb found in home gardens and roadsides. Regarding their growth form, 75% are aquatic herbs such as *Hydrolea zeylanica*, while 25% are terrestrial herbs such as *Oenanthe javanica*.

6. Thirteen percent of the plants grow on roadsides. No plants were exclusive to roadsides. All plants found at roadsides also grow in home gardens. Nine roadside plant species also occur in the rice fields, seven in the upland plantations and six in the woody areas. Most of the wild food roadside plant species were trees such as *Pithecellobium dulce *and *Cassia siamea*. There are a few climbers such as *Passiflora foetida*.

Wild food plants are widely distributed in the anthropogenic landscape. The results show that 80% of the wild food plants can be found in multiple growth locations, particularly rice fields, woods and home gardens. Forty percent of the wild food plants we documented grow in two different locations, 24% grow in three locations and 16% grow in four or more different locations. This can be explained, in part, by species being moved from one place to another facilitated by different degrees of management. This is consistent with the findings of Price [14] and Chanaboon et. al. [46], who reported the presence of wild food plant management practices in Northeast Thailand.

**Figure 2:**
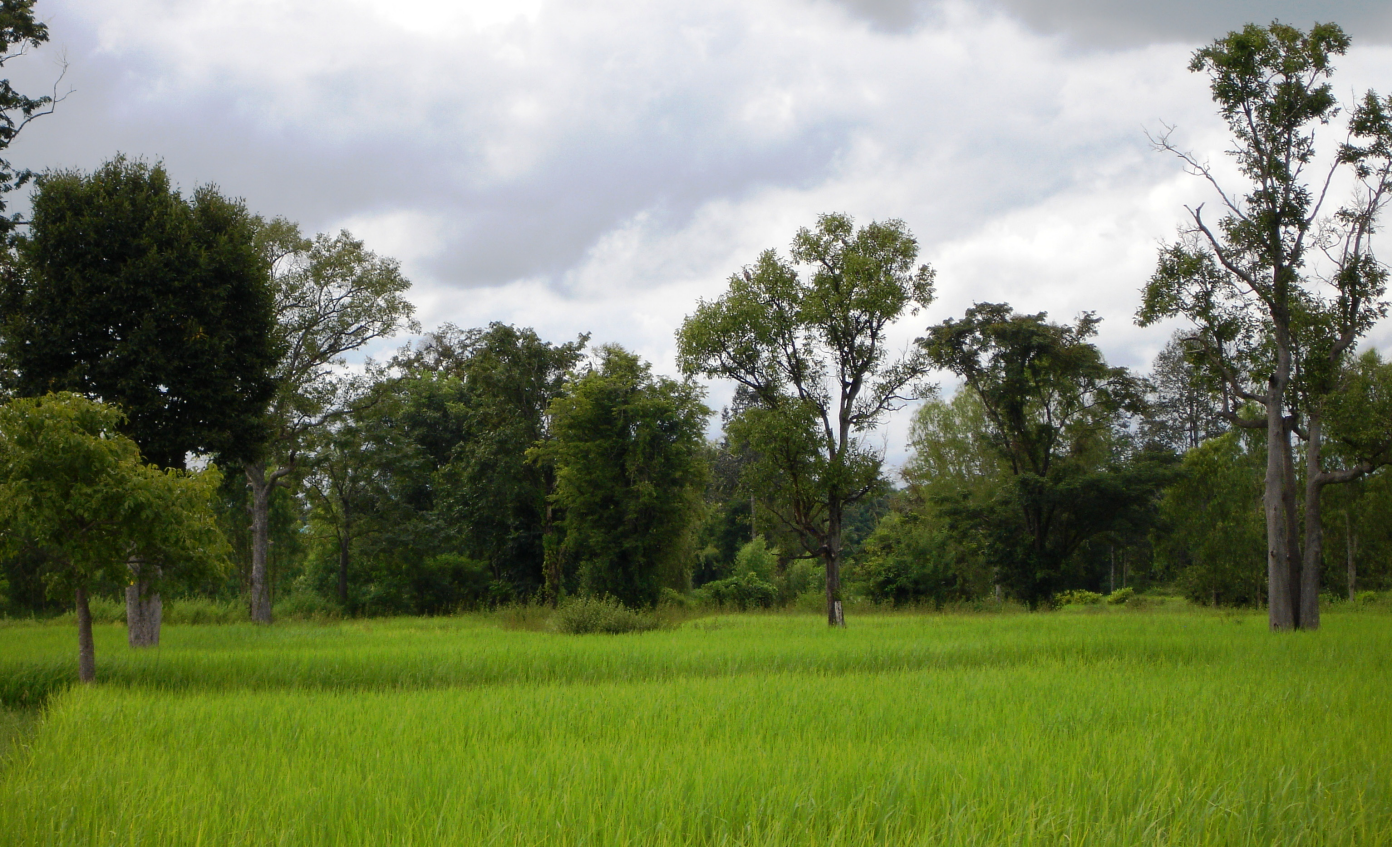
**The presence of trees characterizes the rice fields in Northeast Thailand**.

Out of the 38 tree species, 31 (82%) are to be found in the secondary woody areas, 26 (68%) in the rice fields, 22 (58%) in home gardens and 22 (58%) in upland fields. As discussed in the introductory section, the presence of trees is a common characteristic of rice ecosystems in Northeast Thailand. Trees grow in hillocks, shelters, tree rows and pond margins diversifying the habitats and facilitating the presence of climbers and other plants in the fields (Figure 2). Most trees are maintained in paddies due to their use value [9]. For instance, two thirds of the trees are medicinal (66%) and, in addition, some provided timber and fuel.

### Multiplicity of uses, including parts used

The edible parts of wild food plants vary from reproductive structures (flowers, fruits, seeds) to vegetative organs (leaves, shoots, stalks of flower, stems and sometimes the whole aerial part is consumed). For somewhat less than half of the plants only one part is edible (47%), e.g. only the shoots of *Neptunia oleracea *are consumed. More specifically, for 25% of the plants two parts are eaten, which is the case of *Adenia viridiflora *(shoot and fruit). For 12%, three parts are eaten, such as *Senna sophera *(shoot, flower and fruit). And for 16% of the plants, more than three parts are eaten as for *Limnocharis flava *(shoot, flower, stalk of flower and fruit).

**Figure 3:**
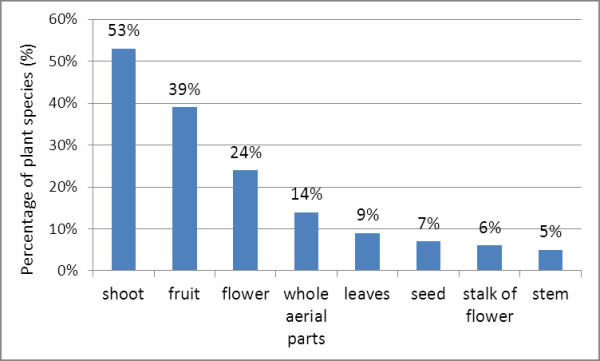
**Edible parts of wild food plants**.

In order to facilitate the analysis, eight categories of different parts consumed were established (see Figure 3):

1. Young shoots sprouting from roots, stems or tips of plants are consumed in 53% of the wild food plants, such as *Bambusa bambos, Senna sophera *and *Telosma minor*. Shoots are widely consumed regardless of the growth form and life cycle of the plant.

2. Fruits, which can be eaten unripe and/or ripe depending on the plant, are consumed in 39% of plants, mainly trees and climbers. The fruit of *Tamarindus indica *is very popular both unripe (it is sour, seasoned with fish sauce and chili) and ripe (it is very sweet, eaten raw or its juice added to a dish of food).

3. Flowers or inflorescences are consumed for 24% of plants. Typical species are *Dolichandrone serrulata *and *Curcuma singularis*.

4. Whole aerial parts, including shoots, young leaves and tender stems, are consumed for 14% of plants. This is the case of many terrestrial and aquatic herbs including *Limnophila aromatica *and *Glinus oppositifolius*, with the exception of *Cuscuta chinensis *that is a climber.

5. Leaves, mainly eaten when young and tender as a raw vegetable or cooked in traditional dishes, are consumed for 9% of plant species like the climber *Cassytha filiformis *and the tree *Leucaena leucocephala*.

6. Seeds are consumed for 7% of plants. For example, the seeds of *Irvingia malayana *are eaten roasted as a snack.

7. Stalks of flower or inflorescence are eaten in the case of 6% of the plants, including *Nymphaea pubescens *whose stalk is eaten raw as a side dish.

8. Stems are consumed for 5% of the plants, including the edible stems of the aquatic herb *Ludwigia adscendens*, the inner core of the trunk of the tree *Borassus flabellifer *(used to make sweets), and the rhizomes of the terrestrial herb *Alpinia malaccensis*.

**Figure 4:**
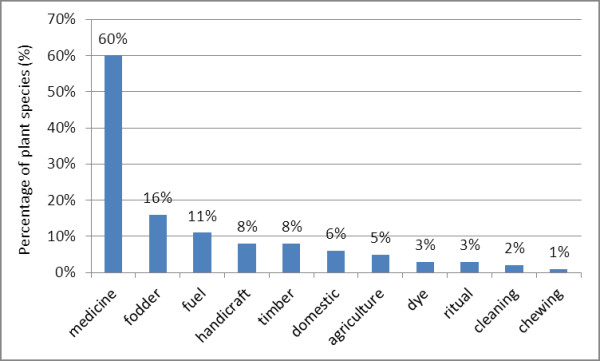
**Additional uses of wild food plants**.

More than two thirds of the wild food plants presented other uses besides food (71%). Some 35% of plants had one additional use, while 26% of the plants had two additional uses, 7% had three additional uses, and three plants had four or more additional uses (see Figure 4).

1. Medicine was the most widely mentioned additional use (60% of the plants). Moreover, it is remarkable that out of the 30 plants with an additional use, 28 have medicinal uses. Some examples of medicinal plants are the herbs *Centella asiatica *and *Ludwigia adscendens*.

2. Fodder use was reported for 16% of the plants. More than half of these fodder plants (9 plants) are also regarded as medicine, such as *Leucaena leucocephala *and *Coccinia grandis*. Fodder plants are mostly herbs, trees and bamboos.

3. Twelve percent of the plants are used as fuel, like *Nephelium hypoleucum *and *Cratoxylum formosum*. Plants used as fuel were mainly trees growing in the rice fields and home gardens, many of them are also found in the woody areas.

4. Timber was reported for 8% of plants. It included trees such as *Xylia xylocarpa *and *Spondias pinnata*.

5. Eight percent of the plants are used for making local handicrafts. The three bamboo plant species are typically used in handicraft production such as in weaving hang mats. The wood of *Artocarpus lacucha *is used to make a traditional musical instrument similar to a xylophone called *pong lang*, which is regarded as the symbol of Kalasin Province.

6. Domestic use was reported for 6% of plants. For example the rattan *Calamus *sp. is used for making home utensils.

7. Five percent of the plants have auxiliary uses. The leaves of *Azadirachta indica *are utilized to make natural insecticide. *Leucaena leucocephala *(Leguminoseae) is used as fertilizer. All four plant species are also used as medicine.

8. Ritual use was reported for 3% of the plants. The Buddhist monks spread holy water using the leaves of *Phyllanthus acidus*. Villagers make curry with the young leaves of *Aegle marmelos *and give it to the monks in blessing ceremonies.

9. Dye was mentioned for 3% of plants used as natural colorants. The fruit of *Tamarindus indica *is used as dye for fish nets. The bark of *Cratoxylum formosum *is utilized to dye clothing.

10. Two plants are used for cleaning, for example *Cassia siamea *is used for making shampoo.

11. Only one plant is used for chewing. The bark of *Artocarpus lacucha *is chewed, sometimes with betle nut.

Consistent with the findings of Price [13] for Northeast Thailand, the importance of wild food plants as food-medicines is present in the current findings. The results indicate that these wild food-medicine plants are important not only for their curative properties, but also for their nutritional and preventive properties. Indeed, this overlapping role as a source of both food and medicine has been documented for farmers' use of wild plants in numerous parts of the world. For example in Vietnam [6], among the Hausa of Northern Nigeria [67], among Albanians and Southern Italians in Lucania [68], in the North West Bank, Palestine [69], and in the Inner Mongolian Autonomous Region, China [70]. Furthermore, undoubtedly, there is an overlap of food, medicine and animal feed, given that almost two thirds of fodder plants are also medicinal (9 out of 14 fodder plants). These results seem to follow the pattern of Ogle et. al. [6] who discussed the multiple functions of wild food plants in Vietnam.

Villagers also mentioned additional uses of wild food plants related to the ecological services they provide. For instance, they commented that the aquatic herb *Monochoria hastata*, which is regarded as a weed of rice fields, provides shade for fish. Additionally, many trees were acknowledged as habitats of red ants and other edible insects. Fish and insects, among other animals, are also gathered from the rice fields constituting an important part of the local diet.

Growth form, growth location, edible parts and additional uses of wild food plants are presented in Table 2.

**Table 2:** Growth form, life cycle, growth location, edible parts and additional uses of wild food plants.

Scientific name	Growth form/Life cycle	Growth location(s)	Edible part(s)	Additional use(s)
**Aizoaceae**				

*Glinus oppositifolius *(L.) Aug.DC.	terrestrial herb/A	rice field	whole aerial parts	

**Amaranthaceae**				

*Amaranthus viridis *L.	terrestrial herb/A	rice field, home garden	shoot, whole aerial parts	medicine, fodder

**Anacardiaceae**				

*Mangifera caloneura *Kurz	tree/P	woods, upland fields	fruit	timber, domestic

*Spondias pinnata *Kurz	tree/P	rice field, home garden, woods, upland fields, roadside	leaves, fruit	medicine, timber

**Annonaceae**				

*Polyalthia debilis *Finet & Gagnep.	shrub/P	home garden, woods, upland fields	fruit	medicine

*Polyalthia evecta *Finet & Gagnep.	tree/P	home garden, woods, upland fields	fruit	medicine

*Uvaria pierrei *Finet & Gagnep.	climber/P	home garden, woods	fruit	

**Araceae**				

*Amorphophallus *sp.	terrestrial herb/A	rice field, woods	shoot	

**Araliaceae**				

*Irvingia malayana *Oliver	tree/P	rice field, woods	seed	medicine, timber, fuel, fodder

**Arecaceae**				

*Borassus flabellifer *L.	tree/P	rice field, home garden, upland fields	flower, fruit, stem	medicine, handicraft

*Calamus *sp.	rattan/P	rice field, home garden, upland fields	shoot, fruit	domestic

**Asclepiadaceae**				

*Telosma minor *Craib	climber/P	rice field, home garden, woods	shoot, flower, fruit	medicine

**Basellaceae**				

*Basella rubra *L.	climber/P	rice field, home garden, woods, upland fields	shoot	medicine

**Bignoniaceae**				

*Dolichandrone serrulata *Seem.	tree/P	woods	flower	medicine

*Oroxylum indicum *Vent.	tree/P	rice field, home garden, woods, upland fields	shoot, flower, fruit	medicine

**Burseraceae**				

*Canarium subulatum *Guillaumin	tree/P	woods	seed	medicine, fuel, fodder

**Campanulaceae**				

*Lobelia begonifolia *Wall.	terrestrial herb/A	rice field	whole aerial parts	

*Lobelia *sp.	terrestrial herb/A	rice field	whole aerial parts	

**Clusiaceae**				

*Cratoxylum formosum *(Jack) Benth. & Hook.f. ex Dyer	tree/P	rice field, home garden, woods	shoot, leaves, flower	fuel, domestic, dye

*Garcinia cowa *Roxb.	tree/P	rice field, woods	shoot, fruit	

**Compositae**				

*Blumea balsamifera *DC.	terrestrial herb/P	rice field	shoot	medicine, ritual

*Emilia sonchifolia *(L.) DC.	terrestrial herb/A	rice field	whole aerial parts	

**Convolvulaceae**				

*Cuscuta chinensis *Lam.	climber/A	rice field, home garden, roadside	whole aerial parts	

*Ipomoea aquatica *Forssk.	terrestrial herb/P	roadside	shoot	medicine, fodder

**Cucurbitaceae**				

*Coccinia grandis *(L.) Voigt	climber/P	home garden, roadside	shoot, flower, fruit	medicine, fodder

*Momordica charantia *L.	climber/A	rice field, home garden	shoot, fruit	medicine

**Ebenaceae**				

*Diospyros rhodocalyx *Kurz	tree/P	rice field, home garden	fruit	medicine

**Euphorbiaceae**				

*Phyllanthus acidus *(L.) Skeels	tree/P	rice field, home garden	shoot, fruit	medicine, ritual

**Fagaceae**				

*Castanopsis *sp.	tree/P	woods	seed	medicine, fuel

**Gnetaceae**				

*Gnetum *sp.	tree/P	rice field, woods	seed	

**Hydrocharitaceae**				

*Ottelia alismoides *(L.) Pers.	aquatic herb/P	rice field, swamps	whole aerial parts	

**Hydrophyllaceae**				

*Hydrolea zeylanica *(L.) J.Vahl	aquatic herb/A	rice field, swamps	shoot, flower	medicine

**Lauraceae**				

*Cassytha filiformis *L.	climber/P	rice field, home garden	leaves, flower, stalk of flower, stem	

**Lecythidaceae**				

*Barringtonia acutangula *(L.) Gaertn.	tree/P	rice field, home garden	shoot, flower	

*Careya arborea *Roxb.	tree/P	woods, upland fields	shoot, flower	

**Leguminosae**				

*Adenanthera pavonina *L.	tree/P	home garden, woods	shoot, flower	

*Cajanus cajan *(L.) Millsp.	shrub/P	rice field, home garden, upland fields	seed	medicine

*Cassia siamea *Lam.	tree/P	rice field, home garden, woods, upland fields, roadside	shoot	medicine, cleaning

*Dialium cochinchinense *Pierre	tree/P	woods, upland fields	fruit	medicine, domestic

*Leucaena leucocephala *(Lam.) de Wit	tree/P	rice field, home garden, woods, upland fields, roadside	shoot, leaves, fruit	medicine, fuel, fodder, auxiliary

*Neptunia javanica *Miq.	terrestrial herb/P	home garden, roadside, swamps	shoot	

*Neptunia oleracea *Lour.	aquatic herb/p	rice field, swamps	shoot	

*Pithecellobium dulce *(Roxb.) Benth.	tree/P	rice field, home garden, roadside	fruit	fuel

*Senna sophera *(L.) Roxb.	shrub/P	rice field, home garden	shoot, flower, fruit	medicine

*Sindora siamensis *Teijsm. ex Miq.	tree/P	rice field, woods, upland fields	fruit	medicine

*Tamarindus indica *L.	tree/P	rice field, home garden, woods, upland fields, roadside	shoot, fruit	medicine, timber, fuel, fodder, dye, cleaning

*Xylia xylocarpa *Taub. var *kerrii *(Craib & Hutch) I.C. Nielsen	tree/P	rice field, woods, upland fields	seed	medicine, timber

**Liliaceae**				

*Asparagus racemosus *Willd.	terrestrial herb/P	rice field, home garden, woods, upland fields	shoot	

**Limnocharitaceae**				

*Limnocharis flava *Buchenau	aquatic herb/A	rice field, swamps	shoot, flower, stalk of flower, fruit	

**Marsileaceae**				

*Marsilea crenata *C.Presl	aquatic herb/P	rice field	whole aerial parts	medicine

**Meliaceae**				

*Azadirachta indica *A.Juss. var. *indica*	tree/P	rice field, home garden, woods, upland fields	shoot, flower	medicine, auxiliary

*Azadirachta indica *A.Juss. var. *siamensis *Valeton	tree/P	rice field, home garden	shoot, flower	medicine, timber, auxiliary

**Menispermaceae**				

*Cissampelos pareira *L.	climber/P	home garden, woods, upland fields	shoot, leaves	medicine

*Tiliacora triandra *Diels	climber/P	home garden, woods, upland fields	shoot, leaves	medicine, domestic

**Menyanthaceae**				

*Nymphoides indica *(L.) Kuntze	aquatic herb/P	swamps	shoot	

**Moraceae**				

*Artocarpus lacucha *Roxb.	tree/P	rice field, woods, upland fields	fruit	medicine, handicraft, chewing

**Myrtaceae**				

*Psidium guajava *L.	tree/P	rice field, home garden, woods, upland fields, roadside	fruit	medicine

*Syzygium cumini *(L.) Skeels	tree/P	rice field, home garden, woods, upland fields	fruit	medicine

*Syzygium gratum *(Wight) S.N.Mitra	tree/P	rice field, home garden, woods, upland fields	shoot, fruit	

**Nymphaeaceae**				

*Nymphaea pubescens *Willd.	aquatic herb/P	rice field, swamps	stalk of flower	medicine

**Onagraceae**				

*Ludwigia adscendens *(L.) H.Hara	aquatic herb/A	rice field, swamps	shoot, leaves, stem	medicine, fodder

**Opiliaceae**				

*Melientha suavis *Pierre	tree/P	woods	shoot, flower	

**Passifloraceae**				

*Adenia viridiflora *Craib	climber/A	woods, upland fields	shoot, fruit	medicine

*Passiflora foetida *L.	climber/A	rice field, home garden, upland fields, roadside	shoot, fruit	

**Poaceae**				

*Bambusa *sp.	bamboo/P	rice field, home garden, woods	shoot	fuel, handicraft, fodder

*Bambusa bambos *(L.) Voss	bamboo/P	rice field, woods, upland fields	shoot	handicraft, fodder

*Vietnamosasa ciliata *(A.Camus) T.Q.Nguyen	bamboo P	woods	shoot	handicraft, fodder

**Pontederiaceae**				

*Eichhornia crassipes *(Mart.) Solms	aquatic herb/P	rice field, swamps	shoot, flower	handicraft, fodder

*Monochoria hastata *(L.) Solms	aquatic herb/A-P	rice field, swamps	shoot, flower, stalk of flower	handicraft, fodder

*Monochoria vaginalis *C.Presl	aquatic herb/A-P	rice field, swamps	whole aerial parts	medicine

**Rhamnaceae**				

*Ziziphus mauritiana *Lam.	tree/P	rice field, home garden, woods, upland fields, roadside	fruit	timber, fuel, dye

*Ziziphus oenoplia *(L.) Mill.	shrub/P	rice field, home garden, woods	fruit	medicine

**Rubiaceae**				

*Oxyceros horridus *Lour.	shrub/P	home garden, woods	shoot, leaves	medicine

*Rothmannia wittii *(Craib) Bremek.	tree/P	woods	fruit	

**Rutaceae**				

*Aegle marmelos *Corrêa	tree/P	rice field, home garden, woods, upland fields	shoot, fruit	medicine, ritual

**Sapindaceae**				

*Lepisanthes rubiginosa *(Roxb.) Leenh.	tree/P	rice field, home garden, woods	fruit	medicine

*Nephelium hypoleucum *Kurz	tree/P	home garden, woods, upland fields	fruit	medicine, fuel

*Schleichera oleosa *(Lour.) Oken	tree/P	rice field, upland fields	fruit	

**Scrophulariaceae**				

*Limnophila aromatica *Merr.	terrestrial herb/A	rice field, home garden	whole aerial parts	medicine

**Umbelliferae**				

*Centella asiatica *(L.) Urb.	terrestrial herb/P	rice field, home garden	whole aerial parts	medicine

*Oenanthe javanica *DC.	terrestrial herb/P	swamps	shoot	

**Zingiberaceae**				

*Alpinia malaccensis *C.Presl	terrestrial herb/P	rice field, woods	shoot, flower, stem	medicine, auxiliary

*Curcuma singularis *Gagnep.	terrestrial herb/P	woods	flower	medicine

*Curcuma *sp.	terrestrial herb/P	woods	flower, stalk of flower	medicine

**Zygnemataceae**				

*Spirogyra *sp.	aquatic herb/A	rice field, swamps	whole aerial parts	medicine

**Unidentified**				

sp. 1	tree/P	woods, upland fields	shoot	medicine

sp. 2	aquatic herb/A	swamps	shoot	medicine, fodder

For life cycle P is perennial and A is annual.

## Conclusions

This study shows the remarkable importance of anthropogenic ecosystems in providing wild food plants. This is reflected in the great diversity of plants found, contributing to the food and nutritional security of rice farmers in Kalasin, Northeast Thailand. The data compiled in this study shows that the majority of wild food plants grow in the different aquatic, semi-aquatic and terrestrial sub-systems offered by rice agro-ecosystems. Trees presented more plants than other growth forms, constituting an important feature of different terrestrial sub-systems of the paddies, such as hillocks, tree rows and shelters. Many important plants are aquatic and terrestrial herbs, as well as climbers. Both annual and perennial species are present in significant numbers.

One of the main findings is that most wild food plants are found in multiple locations, and more than half of them grow either in rice fields and home gardens, rice fields and woods, home gardens and woods, or rice fields, home gardens and woods. No plants were exclusive to home gardens and very few plants were exclusive to woods and rice fields. From these results we assert that farmers play an active role in managing many of these plants, for example, transplanting them from the woods to the fields or to home gardens, making them available in those anthropogenic places located closer to their house and village. This assertion follows the patterns proposed by the "botanical dietary paradox", which clarifies the use of so many wild food plants by farmers in that when deforestation occurs, farmers change to gathering new wild food plants closer to home, including the weeds of agriculture [8,38].

Another major point to note from the results of this research is that more than half of the wild food plants have many edible parts, and more than two thirds of them have additional uses. Shoots, sprouting from the tips of plants, stems or roots, were the most widely cited as consumed regardless of the growth form or life cycle of the plant. Fruits were also common, particularly collected from trees and climbers. Wild food plants presented more than eleven additional uses, accentuating their overall relevance for rice farmers. The most common additional use was for medicine.

The data compiled in this study highlights the necessity to better understand the role of anthropogenic ecosystems in providing wild food plant resources. Further research needs to be carried out on the seasonal quantification of their environmental availability, as well as the location of actual gathering events. Finally, research on transplanting and other management practices would allow us to better comprehend the distribution of these plants in the different ecosystems.

## Competing interests

The authors declare that they have no competing interests.

## Authors' contributions

GCG and LP designed, conducted data collection and interpreted the data. GCG performed the data processing and analysis. The taxonomic revision was coordinated by GCG and LP Literature retrieval was done by both authors. GCG drafted the manuscript, which was revised by LP All authors read and approved the final manuscript.
